# Extracellular Production, Characterization, and Engineering of a Polyextremotolerant Subtilisin-Like Protease From Feather-Degrading *Thermoactinomyces vulgaris* Strain CDF

**DOI:** 10.3389/fmicb.2020.605771

**Published:** 2020-12-21

**Authors:** Yidi Ding, Yong Yang, Yuxia Ren, Jingying Xia, Feng Liu, Yu Li, Xiao-Feng Tang, Bing Tang

**Affiliations:** ^1^State Key Laboratory of Virology, College of Life Sciences, Wuhan University, Wuhan, China; ^2^Hubei Provincial Cooperative Innovation Center of Industrial Fermentation, Wuhan, China

**Keywords:** serine protease, thermostability, halotolerance, organic solvent, detergent, keratinase

## Abstract

Here, the gene encoding a subtilisin-like protease (protease Als) was cloned from *Thermoactinomyces vulgaris* strain CDF and expressed in *Escherichia coli*. The recombinant enzyme was released into the culture medium of *E. coli* as a mature form (mAls). Purified mAls displayed optimal activity at 60–70°C and pH 10.0 using azo-casein as the substrate, and showed a half-life of 13.8 h at 70°C. Moreover, the activity of thermostable mAls was comparable to or higher than those of mesophilic subtilisin Carlsberg and proteinase K at low temperatures (10–30°C). Protease Als was also stable in several organic solvents and showed high compatibility with commercial laundry detergents. Notably, mAls exhibited approximately 100% of its activity at 3 M NaCl, and showed enhanced thermostability with the increase of NaCl concentration up to 3 M. Protease Als possesses an excess of solvent-accessible acidic amino acid residues, which may account for the high halotolerance of the enzyme. Compared with homologous protease C2 from the same strain, protease Als exhibits substantially lower activity toward insoluble keratin substrates but efficiently hydrolyzes soluble keratin released from chicken feathers. Additionally, direct substitution of the substrate-binding site of protease Als with that of protease C2 improves its activity against insoluble keratin substrates. By virtue of its polyextremotolerant attribute and kerationolytic capacity, protease Als may find broad applications in various industries such as laundry detergents, food processing, non-aqueous biocatalysis, and feather processing.

## Introduction

The members of subtilisin-like serine proteases (subtilases) superfamily are widely distributed in bacteria, archaea, and eukaryotes, and contribute to important biological processes such as protein metabolism, nutrition, protein processing, and pathogen invasion (Siezen and Leunissen, 1997; Rao et al., 1998). Subtilases have been extensively studied not only to provide insight into the mechanism of enzyme catalysis and the structure-function relationship of proteins, but also because of their significant applications in detergents, leather processing, food and medicine fields (Gupta et al., 2002). In addition, many subtilases are able to hydrolyze keratin, which has a highly rigid structure rendered by extensive cross-linkages of disulfide bonds and is resistant to hydrolysis by commonly known proteases like trypsin and pepsin. Microbial/enzymatic degradation is regarded as an environmentally friendly approach to recycle keratin-containing wastes from poultry and leather industries (Gupta and Ramnani, 2006), and some keratinolytic microorganisms and proteases have been patented (Shih and William, 1992; Burtt and Ichida, 1999).

Subtilases from extremophiles have attracted increasing attention as promising materials for understanding the molecular basis of protein adaptation to harsh environments including extreme temperature, pH, and salt concentration etc., and studying these enzymes also greatly expands the reaction conditions of biocatalysis (Atomi et al., 2011; Elleuche et al., 2015; Salwan and Sharma, 2019). Thermostability is one of the main requirements for industrial enzymes since thermal inactivation represents a common problem in the application of biocatalysts. Meanwhile, protein substrates generally tend to be disordered at high temperatures and under highly alkaline conditions, rendering them more sensitive to proteolysis. Thermophile-derived thermostable subtilases with highly alkaline pH optima are highly desired in detergent and leather industries, and show great potential in the degradation of insoluble and hard-to-degrade animal proteins such as collagen, keratin, and prion proteins (Jang et al., 2002; Suzuki et al., 2006; Salwan and Sharma, 2019). In addition, subtilases from halophilic/halotolerant microorganisms usually function at high salt concentrations and could occasionally display polyextremotolerant attributes like tolerance to alkaline pH, elevated temperature, and organic solvent etc. (Mokashe et al., 2018); nevertheless, they are less thermostable than their counterparts from thermophiles. The exploration of more robust subtilases with polyextremotolerant attributes is not only scientifically significant for further understanding the mechanism of enzyme adaptation, but it is also of great practical importance in developing proteolytic biocatalysts with a wider versatility to multiple extreme conditions commonly encountered in industrial applications.

*Thermoactinomyces* species generally flourish in decaying hay, compost, and other high-temperature habitats, and have been used to produce a variety of thermostable proteases including metalloproteinase (Georgieva et al., 2000; Zabolotskaya et al., 2004; Majumder et al., 2013), carboxypeptidase (Akparov et al., 2015), glutamyl endopeptidase (Demidyuk et al., 1997), collagenase (Petrova et al., 2006), keratinase (Ignatova et al., 1999; Verma et al., 2016; Wang et al., 2019), and alkaline serine protease (Teplyakov et al., 1990; Gros et al., 1991; Tsuchiya et al., 1992, 1997; Lee et al., 1996). Among these enzymes, thermitase, the well-known alkaline serine protease from *Thermoactinomyces vulgaris*, serves as a model for studying enzyme structure–function relationship (Siezen and Leunissen, 1997). The genes encoding extracellular alkaline serine proteases from *Thermoactinomyces* sp. E79 (Lee et al., 1996) and *Thermoactinomyces* sp. YT06 (Wang et al., 2019) have been determined, showing that they belong to the thermitase family of subtilases. *T. vulgaris* strain CDF is capable of degrading chicken feathers at high temperatures (Cheng et al., 2009; Wang et al., 2015). A spore-associated subtilase (protease CDF) (Cheng et al., 2009), an extracellular subtilase (protease C2) (Wang et al., 2015), and a glutamyl endopeptidase (TS-GSE) (Liu et al., 2016) of the strain CDF have been characterized. The amino acid sequence of protease C2 is identical to that of protease E79 from *Thermoactinomyces* sp. E79 (Lee et al., 1996), albeit significant differences are seen in the upstream flanking regions of each gene. Protease C2 is able to efficiently hydrolyze chicken feathers at high temperatures and under alkaline conditions, representing a promising candidate for enzymatic processing keratinous wastes (Wang et al., 2015). By analyzing the complete genome sequence of the strain CDF (Li et al., 2019), a second gene encoding an extracellular subtilase (named protease Als; GenBank No. QBK13760) with an unusually low isoelectric point (pI) value of 4.26 was identified. In this study, the gene of protease Als was expressed in *Escherichia coli*, and enzymatic properties of the recombinant enzyme, including its tolerances to high temperature, high pH, high salinity, organic solvent, and detergent, were studied. The roles of charged amino acid residues on the enzyme surface in the polyextremotolerant behavior of protease Als were discussed. The capacity of protease Als to hydrolyze chicken feathers at high temperatures was also investigated, and keratinolytic activity of the enzyme was improved by modifying its substrate-binding site.

## Materials and Methods

### Strains and Growth Conditions

*Thermoactinomyces vulgaris* strain CDF was isolated from the campus soil of Wuhan University, China (Cheng et al., 2009), and has been deposited in the China Center for Type Culture Collection (CCTCC) under the accession number AB206328. The strain CDF was grown at 55°C in Luria–Bertani (LB) medium and used for extraction of genomic DNA as described previously (Cheng et al., 2009). *E. coli* DH5α and *E. coli* BL21 (DE3) were used as hosts for cloning and protein expression, respectively, and were grown at 37°C in LB medium containing kanamycin (30 μg/ml) as needed.

### Plasmid Construction and Mutagenesis

The plasmid pET26b (Novagen) was used as the vector for expressing recombinant proteins in *E. coli* BL21 (DE3). The primer sequences and the primer pairs used for PCR were listed in Supplementary Tables 1, 2, respectively. The DNA sequences encoding the protease Als precursor (pre-Als), the signal peptide-lacking proform (pro-Als), and the mature domain (mat-Als) were amplified from the genomic DNA of the strain CDF, and inserted into the *Nde*I-*Eco*RI site of pET26b to construct the expression plasmids pET26b-*pre-Als*, pET26b-*pro-Als*, and pET26b-*mat-Als* for the target proteins, each with a C-terminal 6 × His-tag. Using pET26b-*pre-Als* and pET26b-*mat-Als* as the templates, the expression plasmids for active-site variants of pre-Als (pre-S225A) and mat-Als (mat-S225A) were constructed by replacing the catalytic residue Ser225 with Ala via the QuikChange site-directed mutagenesis method (Papworth et al., 1996). The substrate-binding site variant of protease Als (AS14C) was constructed using the overlapping extension PCR method, as described previously (Bian et al., 2006). All recombinant plasmids were confirmed by DNA sequencing.

### Expression and Purification

*Escherichia coli* BL21(DE3) cells harboring recombinant plasmids were cultured in LB medium, and the expression of recombinant proteins were carried out as described previously (Cheng et al., 2009). After induction with 0.4 mM isopropyl β-D-thiogalactopyranoside (IPTG) for 6 h at 30°C, the *E. coli* cultures were centrifuged at 6,500 × *g* for 10 min at 4°C to separate the culture supernatants and the cells. After washing with buffer A (50 mM Tris–HCl, 10 mM CaCl_2_, pH 8.0), *E. coli* cells were suspended in buffer A, followed by sonication on ice. Soluble and insoluble cellular fractions were separated by centrifugation at 13,400 × *g* for 10 min at 4°C. The recombinant proteins with a C-terminal 6× His-tag in the culture supernatants and the soluble cellular fractions were purified using affinity chromatography on a Ni^2+^-charged Chelating Sepharose^TM^ Fast Flow resin column (GE Healthcare, Little Chalfont, United Kingdom) as described previously (Bian et al., 2006). The purified mature protease C2 was prepared as described previously (Wang et al., 2015). The concentrations of purified enzyme samples were determined using the Bradford assay method (Bradford, 1976) with bovine serum albumin (BSA) as the standard.

### Enzyme Activity Assays

The standard assay for azo-caseinolytic activity of the enzyme was carried out at 60°C for 15 min in 200 μl of reaction mixture containing 20 μl of the enzyme sample and 0.25% (w/v) azo-casein (Sigma, St. Louis, MO, United States) in buffer A. The reaction was terminated by adding 200 μl of 40% (w/v) trichloroacetic acid (TCA). After standing at room temperature (∼25°C) for 15 min, the mixture was centrifuged at 13,400 × *g* for 10 min, and the absorbance of the supernatant at 335 nm was measured in a 1-cm light-path cell. One unit (U) of activity was defined as the amount of enzyme required to increase the corresponding absorbance value by 0.01 unit per minute under the conditions described above.

The proteolytic activity of enzymes on the substrate *N*-succinyl-Ala-Ala-Pro-Phe-*p*-nitroanilide (suc-AAPF-pNA) (Sigma) was measured at the temperatures indicated in buffer A containing 0.1 mM suc-AAPF-pNA. The activity was recorded by monitoring the initial velocity of suc-AAPF-pNA hydrolysis at 410 nm in a thermostated spectrophotometer (SP752; Shanghai Spectrum Instruments Co. Ltd, China). This velocity was calculated on the basis of an extinction coefficient for *p*-nitroaniline (pNA) of 8,480 M/cm at 410 nm (DelMar et al., 1979). One unit (U) of enzyme activity was defined as the amount of enzyme that produced 1 μmol of pNA per minute under assay conditions.

To determine the keratin/casein ratio of the enzyme, the proteolytic activity of the enzyme against keratin (TCI, Japan) or casein (Sigma) was measured as described previously (Jaouadi et al., 2010, 2014) with some modifications. The activity assay was carried out at 60°C for 30 min in 300 μl of reaction mixture containing 50 μl of suitably diluted enzyme solution and 1% (w/v) casein or keratin in buffer A. The reaction was terminated by adding 250 μl of 20% (w/v) TCA. After standing at room temperature (∼25°C) for 30 min, the mixture was centrifuged at 10,000 × *g* for 20 min. Thereafter, 100 μl of the supernatant was mixed with 500 μl of 0.5 M Na_2_CO_3_ and 100 μl of Folin-Ciocalteu’s phenol reagent, followed by standing at room temperature for 30 min. After centrifugation at 10,000 × *g* for 20 min, the absorbance of the resulting supernatant at 660 nm was measured in a 1-cm light-path cell. One unit (U) of activity was defined as the amount of enzyme required to produce 1 μg of amino acid equivalent to tyrosine per minute under the conditions described above.

The proteolytic activity of the enzyme against insoluble substrate [keratin azure or collagen (Sigma)] was determined as described previously (Wang et al., 2015) with some modifications. Insoluble substrates were washed three times using buffer A. A reaction mixture (800 μl) containing 50 μl of the enzyme sample and 3 mg of the substrate in buffer A was incubated at 60°C for 40 min (collagen) or 2 h (keratin azure). The reaction was terminated by cooling on ice. After centrifugation at 13,400 × *g* for 10 min, the absorbance of the supernatant was measured in a 1-cm light-path cell at 595 nm for keratin azure or 280 nm for collagen. One unit (U) of activity was defined as the amount of enzyme required to increase the absorbance at 595 nm (keratin azure) or at 280 nm (collagen) by 0.01 unit per minute under the conditions described above.

### Degradation of Chicken Feather and Soluble Feather Keratin

Enzymatic degradation of chicken feathers was conducted as described by Liang et al. (2010) with some modifications. Briefly, sterilized chicken feather (3 mg) was incubated with purified enzyme (100 μg/ml) at 60°C in buffer A containing 2% β-mercaptoethanol (β-ME). At different time intervals, the disintegration of feather was recorded photographically. The hydrolyzing activity of the enzyme toward soluble feather keratin was conducted as follows. Sterilized chicken feathers were cut into pieces ∼2–3 mm in length and incubated at 60°C for 2 h in buffer A containing 2% β-ME, followed by centrifugation at 13,400 × *g* for 10 min to collect the supernatant containing soluble feather keratin. After the addition of the enzyme (1 μg/ml) into the feather keratin solution, the reaction mixture (150 μl) was incubated at 60°C for 2 h. The reaction was terminated by adding 150 μl of 40% (w/v) TCA, and the precipitated degrading products of feather keratin were subjected to sodium dodecyl sulfate-polyacrylamide gel electrophoresis (SDS-PAGE) analysis.

### Effects of Surfactants, Oxidizing Agent, Organic Solvents and Detergents on Enzyme Activity and Stability

The enzyme (5 μg/ml) was pre-incubated at 40°C for 1 h in buffer A containing different concentrations of surfactants [sodium dodecyl sulfate (SDS), sodium dodecylbenzene sulfonate (SDBS), Triton X-100, Tween 20, and Tween 80], or in buffer B (50 mM Borate-NaOH, 10 mM CaCl_2_, pH 8.0) containing different concentrations of H_2_O_2_. The pre-incubation of the enzyme with different concentrations of organic solvents [methanol, ethanol, isopropanol, acetone, dimethyl sulfoxide (DMSO)] was carried out in buffer A at 40°C for 1 h with shaking (Ibrahim et al., 2015). After incubation, the residual activity of the enzyme was determined by a standard assay for azo-caseinolytic activity at 60°C.

The effect of commercial laundry detergents on enzyme stability was investigated according to the method described by Ibrahim et al. (2015) with minor modification. The commercial laundry detergents include Tide (Procter & Gamble Co.), OMO (Unilever), Liby (Guangzhou Liby Enterprise Group Co.), and Whitecat (Shanghai Hutchison Whitecat Co., Ltd.). The detergent solutions (in tap water) were heat-treated at 90°C for 1 h to inactivate endogenous proteases. The enzyme (5 μg/ml) was pre-incubated at 40°C for 1 h or at 30°C for 24 h in buffer B containing 1.0% of the detergent. Thereafter, the residual activity of the enzyme against *N,N*-dimethylated casein (Sigma) was determined according to the method of Mechri et al. (2019), except that the reaction was carried out at 60°C for 30 min in buffer B.

### Sodium Dodecyl Sulfate-Polyacrylamide Gel Electrophoresis and Immunoblot Analyses

Sodium dodecyl sulfate-polyacrylamide gel electrophoresis was performed using the glycine-Tris buffer (King and Laemmli, 1971) or Tricine-Tris buffer systems (Schägger and von Jagow, 1987). To prevent self-degradation of the protease during sample preparation (boiling) or electrophoresis, the sample was precipitated by the addition of 20% TCA and then washed with acetone before being subjected to SDS-PAGE. The anti-His-tag monoclonal antibody (Novagen) was used for immunoblot analysis, as described previously (Cheng et al., 2009).

## Results

### Extracellular Production of Protease Als in *E. coli*

**FIGURE 1:**
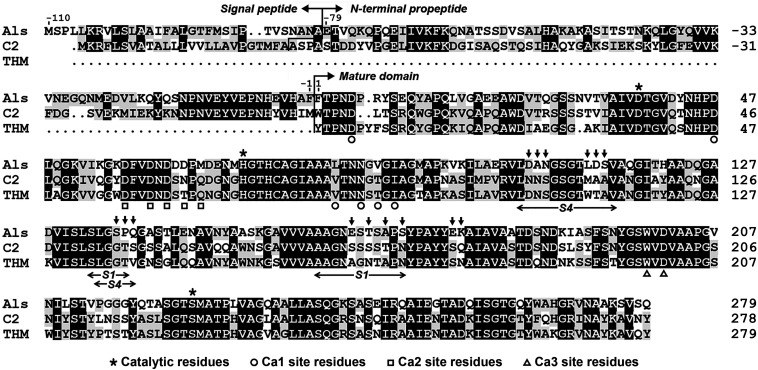
Amino acid sequence alignment of protease Als (QBK13760) with protease C2 (ADD51544) and thermitase (THM, 1105242A). The signal peptide, N-terminal propeptide, mature domain, and the regions of the S1 and S4 pockets of the substrate-binding site are indicated. The catalytic residues and the residues of three Ca^2+^-binding sites (Ca1, Ca2, and Ca3) in thermitase are marked. The residues indicated by vertical arrows above the sequence of protease Als represent those that were replaced by corresponding residues of protease C2 to construct the substrate-binding site variant AS14C. The amino acid residues are numbered starting from the N-terminus of the mature domain.

Sequence alignment analysis shows that protease Als gene encodes a precursor comprising a predicted Sec-type signal peptide of 31 residues, an N-terminal propeptide of 79 residues, and a mature domain of 279 residues (Figure 1). The mature domain of protease Als shares 98.2, 66.8, and 65.6% amino acid sequence identities to protease KERTYT from *Thermoactinomyces* sp. YT06 (Wang et al., 2019), thermitase from *T. vulgaris* (Teplyakov et al., 1990), and protease C2 from *T. vulgaris* strain CDF (Wang et al., 2015), respectively.

**FIGURE 2:**
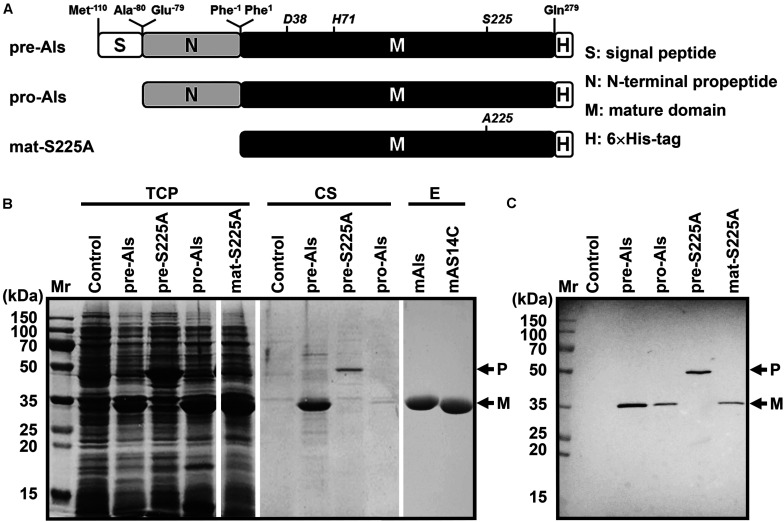
Production and purification of recombinant protease Als and its variants expressed in *E. coli*. **(A)** Schematic representation of the primary structures of protease Als precursor and its derivatives. **(B)** SDS-PAGE analysis of total cellular protein (*TCP*) and culture supernatant (*CS*) of *E. coli* cells expressing recombinant proteins, as well as the purified enzymes (*E*). **(C)** Immunoblot analysis of recombinant proteins in TCP using anti-His-tag monoclonal antibody. The positions of the proform (*P*) and the mature form (*M*) are indicated.

The gene encoding protease Als precursor with a C-terminal 6× His-tag (pre-Als) was cloned and expressed in *E. coli*. For comparison purpose, a signal peptide-lacking proform (pro-Als) and active-site variants of the precursor (pre-S225A) and its mature domain (mat-S225A) were also constructed (Figure 2A). A 34-kDa product was detected in both total cellular protein (TCP) and culture supernatant of *E. coli* expressing pre-Als (Figure 2B). The 34-kDa product displayed the same apparent molecular mass as mat-S225A, and both could be detected using anti-His-tag monoclonal antibody (Figure 2C). These results demonstrate that recombinant pre-Als could be released into the culture medium and converted into its mature form (named mAls) by processing of the N-terminal propeptide. In contrast, pro-Als was detected only in TCP as the 34-kDa mAls (Figure 2B), suggesting that signal peptide is necessary for the release of the enzyme into the culture supernatant and does not prevent the maturation of the enzyme within the cell. Similar to the case of *E. coli* harboring a blank vector (control), no additional host cell proteins were detected in the culture supernatant of *E. coli* expressing pro-Als (Figure 2B), implying that intracellular accumulation of mAls does not cause detectable cell lysis. When pre-S225A was produced in *E. coli*, the recombinant protein with an apparent molecular mass of 45 kDa was also found in both TCP and culture supernatant, but the amount of the active-site variant in the culture supernatant was much lower than that of mAls derived from pre-Als (Figure 2B). These data suggest that the proteolytic activity of protease Als not only mediates the autoprocessing of the N-terminal propeptide but also contributes to the extracellular production of the enzyme in *E. coli*.

### Protease Als Is a Thermostable Alkaline Enzyme With High Low-Temperature activity

**FIGURE 3:**
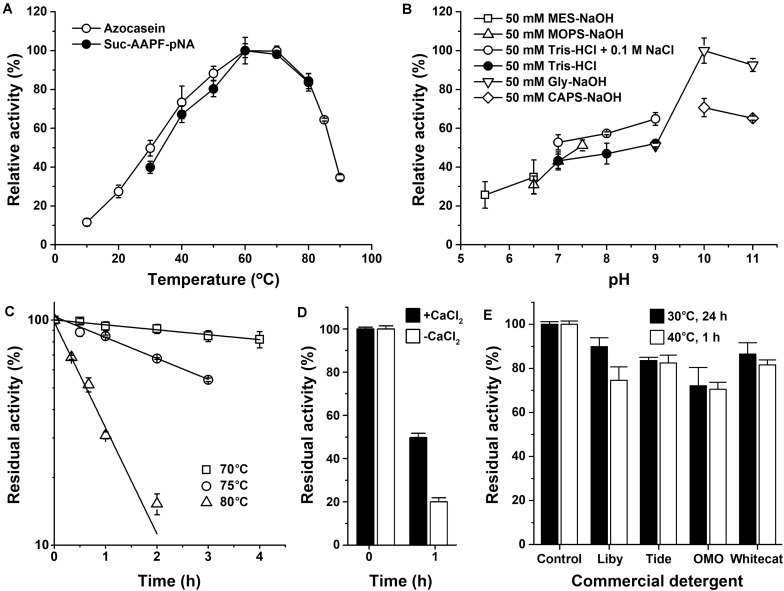
Enzymatic properties of mAls. **(A)** Temperature dependence of proteolytic activity. Activity assays were performed in buffer A (pH 8.0) at the indicated temperatures using 0.25% azo-casein or 0.1 mM suc-AAPF-pNA as the substrates. Relative activity was calculated with the highest level of activity observed at 60°C defined as 100%. **(B)** pH dependence of enzyme activity. The azo-caseinolytic activity of mAls was determined at 60°C in the buffers with different pH values as indicated. The relative activity was calculated with the highest level of activity observed at pH 10 defined as 100%. **(C)** Thermostability of mAls at pH 8.0. The enzyme (1 μg/ml) was incubated in buffer A (50 mM Tris-HCl, 10 mM CaCl_2_, pH 8.0) at different temperatures as indicated. At the time intervals indicated, aliquots were withdrawn and subjected to azo-caseinolytic activity assay at 60°C. The residual activity is expressed as a percentage of the initial activity. **(D)** Thermostability of mAls at pH 10.0. The enzyme (1 μg/ml) was incubated at 60°C for 1 h in 50 mM Glycine-NaOH (pH 10.0) in the absence (−) or presence (+) of 10 mM CaCl_2_ and then subjected to azo-caseinolytic activity assay at 60°C. The residual activity is expressed as a percentage of the initial activity. **(E)** Stability of mAls in commercial laundry detergents. The enzyme (5 μg/ml) was incubated with 1% of each detergent at 40°C for 1 h or at 30°C for 24 h, and then subjected to activity assay at 60°C using N,N-dimethylated casein as the substrate. The residual activity is expressed as a percentage of the activity of the enzyme sample incubated under similar conditions in the absence of detergent. The values are expressed as means ± standard deviations (SDs) of two or three independent experiments performed in triplicate.

The mature protease Als (mAls) with a His-tag at the C terminus was purified by affinity chromatography using a Ni^2+^-charged column (Figure 2B). Using azo-casein or suc-AAPF-pNA as a substrate, the optimum temperature of purified mAls was determined to be 60–70°C at pH 8.0 (Figure 3A). The effect of pH on the enzyme activity was measured at 60°C over a pH range of 5.5–11.0, showing that mAls has an optimum pH of 10.0, with approximately 93% of this activity retained at pH 11.0 (Figure 3B). It was noticed that among the buffers used the Tris–HCl buffer is not supplemented with Na^+^. When 100 mM NaCl was added in Tris–HCl buffers (pH 7.0–9.0), mAls exhibited higher activities (Figure 3B), implying that Na^+^ could promote the activity of the enzyme. In the presence of 10 mM CaCl_2_ at pH 8.0, mAls retained more than 90% of the original activity after incubation at 60°C for 12 h (Supplementary Figure 1), and showed half-lives of 13.8 h, 3.4 h, and 37 min at 70, 75, and 80°C, respectively (Figure 3C). At pH 10.0, mAls retained 20 and 50% of its initial activity after 1 h incubation at 60°C in the absence and presence of 10 mM CaCl_2_, respectively (Figure 3D). These results demonstrate that protease Als is a Ca^2+^-dependent thermostable alkaline subtilase.

**FIGURE 4:**
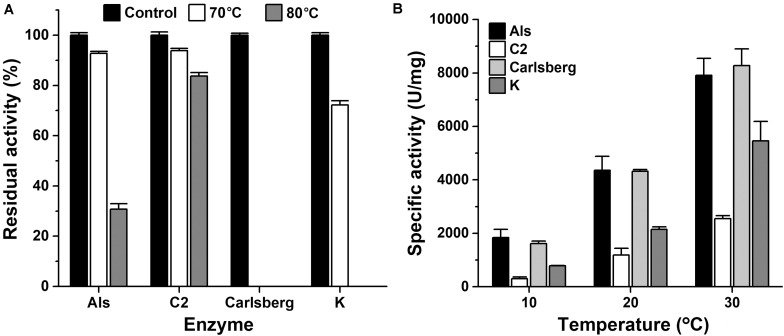
Comparison of thermostabilities and low-temperature activities of mAls, protease C2, subtilisin Carlsberg, and proteinase K. **(A)** Heat inactivation of the enzymes. Each enzyme (1 μg/ml) was incubated at 70 or 80°C in buffer A for 1 h and then subjected to azo-caseinolytic activity assay at 60°C. The residual activity is expressed as a percentage of the initial activity. **(B)** Specific activities of the enzymes at low temperatures. Proteolytic activities of the enzymes were determined in buffer A at the indicated temperatures using 0.25% azo-casein as the substrate. The values are expressed as means ± SDs of three independent experiments performed in triplicate.

The thermostability and low-temperature activity of mAls were compared with those of thermophilic protease C2 and mesophilic subtilases such as subtilisin Carlsberg and proteinase K. After heat treatment at 70 or 80°C for 1 h, mAls retained 93 or 31% of its initial activity, respectively (Figure 4A). In contrast, subtilisin Carlsberg was completely inactivated by heat treatment at 70 or 80°C, while proteinase K retained 72% of its activity after 1 h-incubation at 70°C but was completely inactivated by heat treatment at 80°C (Figure 4A). These results suggest that protease Als is much more thermostable than subtilisin Carlsberg and proteinase K. Although mAls was less resistant to heat treatment at 80°C than protease C2 (Figure 4A), it exhibited a remarkably higher activity than the latter at 10–30°C (Figure 4B). Moreover, the activity of mAls was comparable to or higher than those of commercial subtilisin Carlsberg and proteinase K at the temperature range of 10–30°C (Figure 4B). These results suggest that the thermostable protease Als is a promising candidate for industrial application in a wide temperature range.

### Protease Als Is Resistant to Organic Solvents and Compatible With Commercial Laundry Detergents

The effects of surfactants, oxidizing agent, and organic solvents on the stability of mAls were investigated by determination of the residual activity of the enzyme sample following pre-incubation with the additives at 40°C for 1 h (Table 1). The enzyme was highly stable in the presence of 1.0% non-ionic surfactants (Triton X-100, Tween 20, and Tween 80) or 0.1% anionic surfactants (SDS and SDBS), but it was less stable at 0.5–1.0% concentrations of SDS or SDBS. The enzyme retained 41.4% of its initial activity in the presence 1.0% H_2_O_2_, while a loss of approximately 90% of its activity was observed at 5.0% H_2_O_2_. In all organic solvents (20–40%) tested except isopropanol, mAls displayed approximately 82–134% of its initial activity, showing a high tolerance to organic solvents.

**TABLE 1:** Effects of surfactants, oxidizing agent, and organic solvents on the stability of protease Als.

Agent	Concentration	Residual activity (%)^a^
Control		100.0 ± 1.5
SDS	0.1% (w/v)	104.4 ± 6.4
	0.5% (w/v)	57.7 ± 0.4
	1.0% (w/v)	45.6 ± 4.6
SDBS	0.1% (w/v)	98.4 ± 5.8
	0.5% (w/v)	57.4 ± 7.3
	1.0% (w/v)	22.1 ± 0.2
Triton X-100	1.0% (v/v)	112.8 ± 7.2
Tween 20	1.0% (v/v)	105.8 ± 3.8
Tween 80	1.0% (v/v)	116.2 ± 4.8
H_2_O_2_	1.0% (v/v)	41.4 ± 1.1
	5.0% (v/v)	10.3 ± 0.5
DMSO	20.0% (v/v)	115.2 ± 4.5
	40.0% (v/v)	133.6 ± 3.2
Acetone	20.0% (v/v)	93.7 ± 4.3
	40.0% (v/v)	94.0 ± 1.5
Methanol	20.0% (v/v)	101.0 ± 5.0
	40.0% (v/v)	86.9 ± 2.4
Ethanol	20.0% (v/v)	94.2 ± 2.7
	40.0% (v/v)	81.5 ± 1.1
Isopropanol	20.0% (v/v)	84.3 ± 1.2
	40.0% (v/v)	67.4 ± 1.3

*^a^After pre-incubation of mAls (5 μg/ml) with each agent at 40°C for 1 h, the azo-caseinolytic activity of the enzyme was determined at 60°C under standard assay conditions as described in section “Materials and Methods.” The residual activity was calculated on the basis of the activity of the enzyme sample without pre-treatment (defined as 100%). The values are expressed as means ± standard deviations of three independent measurements.*

The compatibility of mAls with commercial laundry detergents was examined by pre-incubating the enzyme with detergents at 40°C for 1 h or at 30°C for 24 h, followed by activity assay. It was found that mAls retained 70–90% of its activity after incubation with the detergents tested (Figure 3E). The remarkable stability of protease Als in commercial laundry detergents suggests that protease Als has the potential to be used as a detergent additive.

### Protease Als Is Highly Halotolerant

In comparison with its closely related homologous protease C2 and thermitase, a unique feature of protease Als is that it contains a larger number of acidic amino acid residues (19 Asp and 10 Glu), and thus a calculated pI value of 4.26 (Supplementary Table 3). A homology modeling of protease Als revealed that the acidic amino acid residues are distributed on the solvent-accessible surface area of the enzyme (Supplementary Figure 2). The possession of a high content of acidic amino acid residues accessible to solvent is rarely observed in common subtilases but is a typical feature of halophilic subtilases (halolysins) from haloarchaea (Supplementary Table 3; Kamekura et al., 1996; Shi et al., 2006). Based on these observations, and the evidence of increased activity of mAls by 0.1 M NaCl (Figure 3B), the effects of salt concentration on the activity and stability of protease Als were investigated.

**FIGURE 5:**
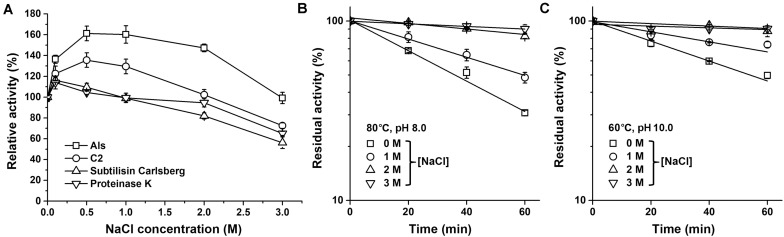
Effects of salt concentration on enzyme activity and stability. **(A)** Salinity dependence of enzyme activity. Using 0.25% azo-casein as the substrate, proteolytic activities of the enzymes (1 μg/ml) were determined at 60°C in buffer A containing different concentrations of NaCl as indicated. Relative activity was calculated by defining the activity of the sample without NaCl as 100%. **(B,C)** Effect of NaCl on thermostability of mAls. The enzyme (1 μg/ml) was incubated at 80°C in buffer A (pH 8.0) **(B)** or at 60°C in 50 mM Glycine-NaOH (pH 10.0) containing 10 mM CaCl_2_) **(C)** in the presence of different concentrations of NaCl. At the time intervals indicated, aliquots were withdrawn and subjected to azo-caseinolytic activity assay at 60°C. The residual activity is expressed as a percentage of the initial activity. The values are expressed as means ± SDs of three independent experiments performed in triplicate.

It was found that the azo-caseinolytic activity of mAls increased by 47–61% in the presence of 0.5–2.0 M NaCl (Figure 5A). The azo-caseinolytic activities of protease C2, subtilisin Carlsberg, and proteinase K were also enhanced in the presence of 0.1–0.5 M NaCl, but to a lesser extent than that of mAls (Figure 5A). Notably, mAls exhibited approximately 100% of its activity at 3 M NaCl, while protease C2, subtilisin Carlsberg, and proteinase K retained 73, 56, and 65% of their activities, respectively, under the same salinity (Figure 5A). At either pH 8.0 or pH 10.0, the half-lives of mAls at 80 or 60°C in the presence of NaCl were longer than that in the absence of NaCl (Figures 5B,C). It is noticed that mAls is very stable at 80°C in the presence of 3 M NaCl, wherein the enzyme maintained more than 90% of its activity after 1-h incubation at pH 8.0 (Figure 5B). These results suggest that NaCl could not only promote the enzymatic activity but also enhance the thermostability of protease Als.

### The Keratinolytic Activity of Protease Als Could Be Improved by Modifying Its Substrate-Binding Region

**FIGURE 6:**
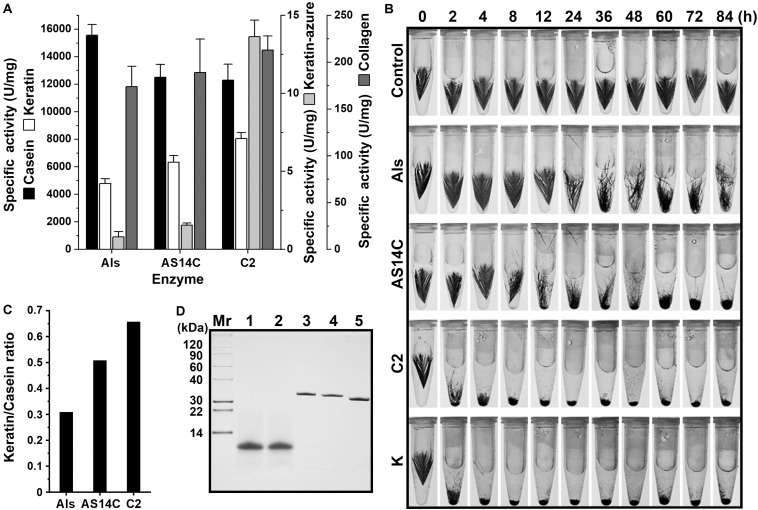
Comparison of the keratinolytic and collagenolytic activities of mAls, mAS14C, and protease C2. **(A)** Specific activities of the enzymes toward casein keratin, collagen, and keratin azure at 60°C. The values are expressed as means ± SDs of three independent experiments performed in triplicate. **(B)** Degradation of chicken feather by the enzymes. Sterilized chicken feathers (3 mg each) were incubated at 60°C in buffer A containing 2% β-ME in the absence (*control*) or presence of each enzyme (100 μg/ml) for different time periods. Proteinase K was used as a reference enzyme. **(C)** Keratin/casein ratios of the enzymes. The keratin/casein ratios were calculated based on the data shown in **(A)**. **(D)** Hydrolysis of soluble feather keratin by the enzymes. Soluble feather keratin (*lane 1*) in buffer A containing 2% β-ME was incubated at 60°C for 2 h in the absence (*lane 2*) or presence of mAls (*lane 3*), AS14C (*lane 4*), or protease C2 (*lane 5*), followed by Tricine-Tris SDS-PAGE analysis.

It was previously found that protease C2 from strain CDF possesses a high keratinolytic activity comparable to that of proteinase K and could hydrolyze collagen at high temperatures (Wang et al., 2015). Here, the keratinolytic and collagenolytic capacities of protease Als from the same strain were investigated. At 60°C, the activity of mAls against keratin azure (α-keratin) was only about 1/20 of that of protease C2, while the collagenolytic activities of the two enzymes were comparable (Figure 6A). Meanwhile, protease C2 and proteinase K [well known for its high keratinolytic activity (Ebeling et al., 1974)] could completely degrade the barbules of chicken feather (β-keratin) within 4 h at 60°C, while mAls only partially disintegrated the barbules after 84 h (Figure 6B), showing that protease Als is a weak insoluble keratin-hydrolyzing enzyme compared to protease C2 and proteinase K. To determine the keratin/casein ratios of protease Als and protease C2, the proteolytic activities of the two enzymes against keratin and casein and were determined, showing that protease Als exhibited a slightly higher caseinolytic activity but a lower keratinolytic activity than protease C2 (Figure 6A). The keratin/casein ratios were calculated to be 0.31 for protease Als and 0.66 for protease C2 (Figure 6C). The hydrolytic activity of mAls toward soluble feather keratin, which was prepared by heat treatment (60°C) of chicken feathers under reducing conditions, was investigated further. The soluble feather keratins released from chicken feathers displayed a molecular mass of about 10 kDa (Figure 6D), in agreement with the theoretical molecular mass of intact chicken keratin (Fraser and Parry, 2011). It was found that both mAls and protease C2 were able to completely degrade the soluble feather keratin (Figure 6D).

When casein and collagen were used as the substrates, mAls and protease C2 exhibited comparable levels of hydrolyzing activities (Figure 6A). It was postulated that the remarkable difference between the two enzymes in their hydrolyzing activities toward insoluble keratin substrates may be due to the difference in substrate preference. To test this possibility, a substrate-binding site variant of protease Als (AS14C) was constructed by substituting its S1 and S4 pockets of the substrate-binding site with those of protease C2, and purified its mature form (mAS14C) (Figure 2B). The variant mAS14C not only showed enhanced activities against keratin substrates (keratin azure and keratin) (Figure 6A) and an increased keratin/casein ratio (Figure 6C), but also disintegrated the barbules of chicken feather more efficiently than protease Als (Figure 6B). These results confirm that the substrate-binding site of protease C2 has a stronger preference for insoluble keratin substrates than that of protease Als. Although mAS14C showed an improved keratinolytic activity, it is still less active than protease C2 in hydrolyzing keratin, keratin azure, and feathers (Figures 6A,B), indicating that, in addition to the substrate-binding site, other parts of enzyme molecule is also important for keratinolytic activity of the enzyme.

## Discussion

Protease Als belongs to the thermitase family of subtilases, and shares high amino acid sequence identity with thermitase (66.8%) and protease C2 (65.6%). Thermitase contains three Ca^2+^-binding sites (Ca1, Ca2, and Ca3) that contribute to its structural stability (Gros et al., 1991). The ligand residues of the three Ca^2+^-binding sites in thermitase are partially (Ca1 and Ca2) or fully (Ca3) conserved in proteases Als (Figure 1). The observed Ca^2+^-dependent thermostability of protease Als confirms that the binding of Ca^2+^ is important for stabilizing the enzyme. In comparison with protease C2, protease Als shows a shorter half-life at high temperatures but is more active at low temperatures. In terms of stability-activity relationship, the behaviors of proteases Als and C2, two homologous enzymes coming from strain CDF, seem to follow the trade-off principle that enzymes can gain higher low-temperature activities by sacrificing their thermostability, and vice versa (Siddiqui and Cavicchioli, 2006). Despite being less stable than protease C2, protease Als is much more thermostable than mesophilic subtilisin Carlsberg and proteinase K. Moreover, protease Als exhibits proteolytic activity comparable to or higher than those of subtilisin Carlsberg and proteinase K at low temperatures. By virtue of its high thermostability and substantial low-temperature activity, protease Als is a promising candidate for practical application in a wide temperature range.

Besides the binding of Ca^2+^, the formation of ion pairs between negatively and positively charged residues on protein surface also contributes to the thermostability of thermophilic proteins (Strickler et al., 2006; Karshikoff et al., 2015). It has been reported that thermitase possesses more surface ion pairs than mesophilic subtilisin BPN’ (Voorhorst et al., 1997). At least eight of the ten surface ion pairs of thermitase are conserved in protease Als, involving six Asp residues, one Glu residue, three Arg residues, and two Lys residues (Supplementary Table 4). The data presented here showed that protease Als is less thermostable but more active at pH 10.0 than at pH 8.0. A possible explanation for this is that the basic residues forming ion pairs tend to be deprotonated at high pH values, and thus the ion-pair interactions important for thermostability would be weakened. Meanwhile, the attenuation of the ion-pair interactions at high pH may confer certain flexibility favorable for enzyme catalysis, reflecting a trade-off between stability and activity (Siddiqui and Cavicchioli, 2006).

Protease Als possesses an excess of solvent-accessible acidic amino acid residues and displays high halotolerance, as evidenced by the findings that the enzyme exhibited improved activity at 0.5-2.0 M NaCl, maintained approximately 100% of its activity at 3 M NaCl, and showed enhanced thermostability with increasing salt concentration. The possession of an excess of acidic amino acid residues located on protein surface is regarded as an adaptive mechanism of halophilic enzymes, wherein the solvent-accessible acidic residues are involved in the formation of a strong hydration shell to maintain structural stability of halophilic enzymes at high salt concentrations (Mevarech et al., 2000; DasSarma and DasSarma, 2015; Mokashe et al., 2018). Meanwhile, halophilic enzymes could maintain their structural flexibility necessary for catalytic activity at high salinity via the electrostatic repulsion of negatively charged residues, while the hydrophobic core in non-halophilic enzymes would be rigidified in the presence of a high concentration of salts, leading to a decrease of the flexibility required for efficient catalysis (Mevarech et al., 2000). Protease Als has such features as halophilic enzymes; nevertheless, it is a halotolerant rather than halophilic enzyme. In contrast to halophilic enzymes, which tend to be destabilized and inactivated at low-salt concentration due to strong repulsion force exerted by the acidic residues (Chakravorty et al., 2017; Mokashe et al., 2018), protease Als is highly stable and active in the absence of NaCl. It was noticed that protease Als possesses a higher content of basic lysine residues compared with homologous halolysins (Supplementary Table 3) which commonly contain few lysine residue along with the excess of acidic residues (Mokashe et al., 2018). The abovementioned surface ion pairs, including those involving lysine residues, may contribute to the stabilization of protease Als in the absence of NaCl. At low salt concentrations, the destabilizing effect of strong electrostatic repulsion by negatively charged residues could be compensated by the stabilizing effect of the ion pairs. At high salt concentrations, both electrostatic repulsion and attraction would be weakened due to increased ionic strength of the solvent, and the enzyme could be stabilized via the formation of a strong hydration shell, mediated by an excess of negatively charged surface residues. Such subtle balance between electrostatic repulsion and attraction plays an important role in the halotolerant behavior of protease Als, allowing it to be stable and active in a wide salinity range.

Some proteases from halophilic/halotolerant microorganisms were reported to be both halotolerant and thermotolerant, but they are generally less stable at high temperatures than their homologs from thermophiles, especially in the absence of salt (Mokashe et al., 2018). Additionally, the information about gene/protein sequences of the reported halo-thermotolerant proteases from halophilic/halotolerant microbes is very limited (Mokashe et al., 2018), thus the structural basis for their polyextremtolerant attributes remains to be elucidated. On the other hand, a common feature of thermophile-derived proteases are their high thermostability, and some of them (e.g., those from marine thermophiles and hyperthermophiles) are resistant to salinity (Barzkar et al., 2018). However, to the best of our knowledge, by far there is no literature showing that a (hyper)thermophile-derived thermostable protease could exhibit 100% of its activity at extremely high salt concentrations (e.g., 3 M NaCl). In the case of protease Als, it showed a half-life of 13.8 h at 70°C in the absence of NaCl, retained more than 90% of its activity after 1-h incubation with 3 M NaCl at 80°C, and exhibited the same level of activity at 3 M NaCl as that in the absence NaCl, thereby representing one of the most robust proteases coupling high thermostability and high halotolerance reported so far. Moreover, the comparative analysis of protease Als with its homologs (e.g., protease C2, thermitase, and halolysins) provides important clues about the roles of surface ion pairs and negatively charged residues in the tolerance of the enzyme to both high temperature and high salinity.

The salt-stable characteristics of halophilic and halotolerant enzymes generally allow these enzymes to be stable and functional in low-water media such as organic solvents (Mokashe et al., 2018). Meanwhile, enzymes from thermophiles are usually reported to be organic solvent-resistant due to their intrinsically stable structures (Sellek and Chaudhuri, 1999). It is not astonishing that protease Als, a thermostable and halotolerant enzyme, is highly resistant to organic solvents. Both thermostable and halophilic/halotolerant proteases have been successfully used for peptide synthesis in the presence of organic solvents (Nagayasu et al., 1994; Ryu et al., 1994; Toplak et al., 2015). Accordingly, the organic solvent-tolerance of protease Als makes the enzyme a potential candidate for use in non-aqueous biocatalysis.

In comparison with protease C2, protease Als exhibited substantially lower hydrolyzing activity toward insoluble keratin substrates such as keratin azure and chicken feathers. Meanwhile, protease Als showed a low keratin/casein ratio (0.31), suggesting that this enzyme is not a true keratinase (Jaouadi et al., 2014) but a subtilase with low keratinolytic activity. Nevertheless, protease Als efficiently hydrolyzes the soluble products released from feathers. Similarly, the recently reported protease KERTYT, which shares 98.2% amino acid sequence identity to protease Als, has the ability to hydrolyze soluble keratin, although it is unclear whether it can degrade insoluble keratin substrates or not (Wang et al., 2019). *T. vulgaris* strain CDF can degrade and grow on chicken feathers not only by secreting keratinolytic proteases but also by providing a supply of reducing power necessary for the cleavage of keratin disulfide bonds (Wang et al., 2015). It seems that both of protease C2 and protease Als are important for strain CDF to use chicken feathers as a nutrient. Protease C2 plays a major role in degrading insoluble keratin substrates, and both protease C2 and protease Als hydrolyze soluble keratins, which may be released from feathers by the action of reducing power of the cells, into smaller peptides available for the assimilation by strain CDF. Notably, substitution of the substrate-binding site of protease Als with that of protease C2 improves the hydrolyzing activity against insoluble keratin substrates and the keratin/casein ratio. Jaouadi et al. (2014) and Fang et al. (2015) reported that a rational design of the substrate-binding region of keratinases could improve the keratinolytic activity of the enzyme. The results presented here suggest that direct replacement of the substrate-binding site of a protease with that of a highly keratinolytic protease is an alternative strategy for engineering of protease with improved keratinolytic activity.

Recombinant protease Als could be released into the culture supernatant of *E. coli* as a mature form. The extracellular production of recombinant proteins in *E. coli* greatly facilitates downstream processing and protein preparation, and is highly desired in industrial applications (Burdette et al., 2018). Proteins released into the culture supernatant of *E. coli* have to pass through both the cytoplasmic and outer membranes. The predicted Sec-type signal peptide of protease Als precursor is necessary for the extracellular release of the enzyme, indicating that recombinant protease Als is translocated across the cytoplasmic membrane via the Sec pathway and secreted into the periplasm. Furthermore, proteolytic activity of protease Als promotes the release of the enzyme into the culture media, implying that the outer membrane permeability of *E. coli* could be improved through the action of the enzyme. It is possible that active mature protease Als in the periplasm could hydrolyze outer membrane proteins, leading to an increase of the outer membrane permeability. Nevertheless, the proteolytic activity is not essential for extracellular release of protease Als, as its active site variant could also be detected in the culture supernatant. The detailed mechanism for the release of recombinant protease Als from the periplasm into the extracellular milieu remains to be further elucidated.

In summary, protease Als from *T. vulgaris* strain CDF is stable and functional under polyextreme conditions. Besides possessing high thermostability and being active at high pH values, it is highly tolerant to high salinity, organic solvents, and detergents. Moreover, the low-temperature activity of protease Als is comparable to or higher than that of some commercial proteases, and the recombinant enzyme could be extracellularly produced by *E. coli*. With these merits, protease Als may find broad applications in laundry detergents, food processing, and non-aqueous biocatalysis, etc. Although protease Als only weakly degrade insoluble keratin substrates, it could efficiently hydrolyze soluble keratin and thus contribute to the utilization of feathers as a nutrient by strain CDF for growth. In addition, the direct replacement of the substrate-binding site by that of a keratinase has been proved to be a valuable method to improve the keratinolytic activity of a protease here. Finally, the excess of acidic amino acid residues and ion pairs on enzyme surface most likely contribute to the high halotolerance and thermostability of protease Als. Future mutational analysis of the surface charged residues of protease Als is warranted to probe the molecular basis for the polyextremotolerant property of the enzyme.

## Data Availability Statement

The datasets presented in this study can be found in online repositories. The names of the repository/repositories and accession number(s) can be found below: https://www.ncbi.nlm.nih.gov/, QBK13760.

## Author Contributions

YD, YY, YR, JX, FL, and YL conducted the experiments. YD, X-FT, and BT analyzed and interpreted the results and contributed to writing the manuscript. All authors contributed to the article and approved the submitted version.

## Conflict of Interest

The authors declare that the research was conducted in the absence of any commercial or financial relationships that could be construed as a potential conflict of interest.
