# Supplementary material for: Extracellular Production, Characterization, and Engineering of a Polyextremotolerant Subtilisin-Like Protease From Feather-Degrading Thermoactinomyces vulgaris Strain CDF
Source: Front Microbiol. 2020 Dec 21;11:605771. doi: 10.3389/fmicb.2020.605771 (PMC7779483; doi:10.3389/fmicb.2020.605771)
Supplement: Supplementary file 1 [file Data_Sheet_1.docx]

Supplementary Material

# Supplementary Figures and Tables

## Supplementary Figures





**Supplementary Figure S1.** Thermostability of mAls at 60°C. The enzyme (1 μg/ml) was incubated at 60°C in buffer A (50 mM Tris-HCl, 10 mM CaCl_2_, pH 8.0). At the time intervals indicated, aliquots were withdrawn and subjected to azocaseinolytic activity assay at 60°C. The residual activity is expressed as a percentage of the initial activity. The values are expressed as means ± standard deviations (SDs) of two independent experiments performed in triplicate.


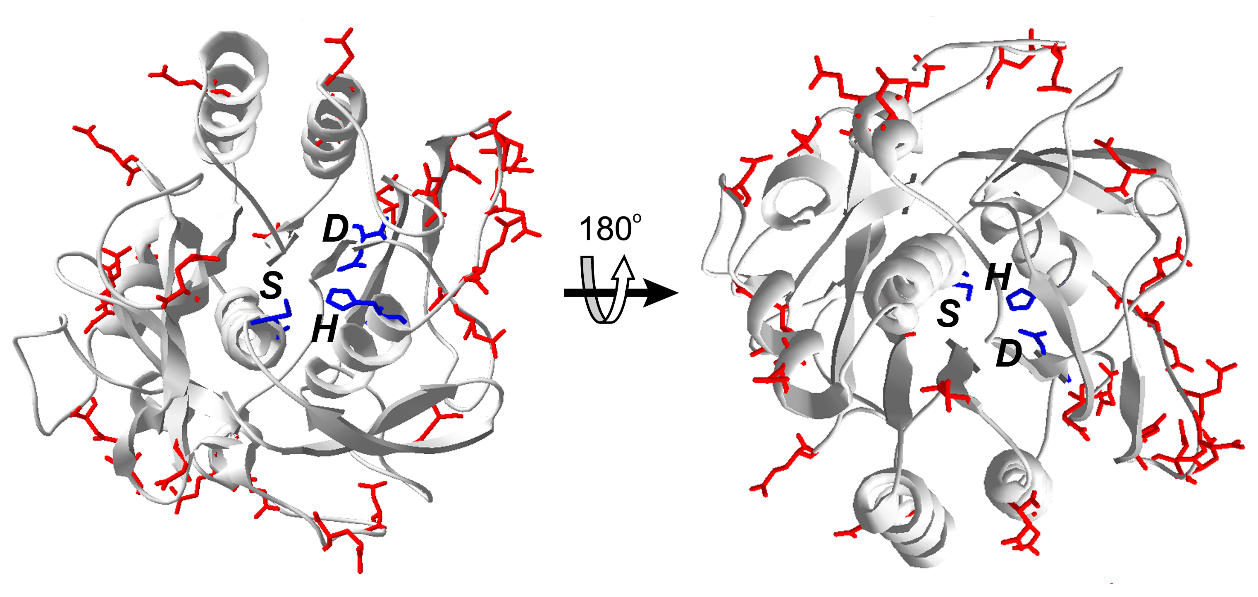


**Supplementary Figure S2.** Distribution of acid amino acid residues of protease Als. The ribbon model of protease Als was generated using SWISS-MODEL (http://swissmodel.expasy.org) with thermitase (PDB ID: 1THM) as the template. The side chains of Asp and Glu (*red*), as well as those of the catalytic triad D-H-S (*blue*) are indicated.

## Supplementary Tables

**Supplementary Table S1. Primers used in this study**

| Primer | Oligonucleotide sequence (5'-3')^a^ |
| --- | --- |
| Als-F | GGAATTCCATATGAGCCCATTGCTGAAACGTGTATTGTC |
| Als-R | CCGGAATTCTTA*GTGGTGGTGGTGGTGGTG*TTGAGAGACAGATTTTGC |
| S225A-F | CGGCTTCCGGAACTGCCATGGCCACACCGCTG |
| S225A-R | CAGCGGTGTGGCCATGGCAGTTCCGGAAGCCG |
| ΔS-5F | GGAATTCCATATGGAGACGGTTCAAAAACAGCCTC |
| M−F | GGAATTCCATATGTTCACTCCGAATGATCC |
| AS1C-F | CAGCCGGGAACTCATCCAGCAGCACTCCCAACTATCCGGCTTATTATTCACAAGCCATCGCAG |
| AS1C-R | CTGCGATGGCTTGTGAATAATAAGCCGGATAGTTGGGAGTGCTGCTGGATGAGTTCCCGGCTG |
| AS4C-F1 | GAACGTGTTTTGAATAACAGCGGATCCGGCACCATGGCTGCCGTTGCACAAG |
| AS4C-R1 | CTTGTGCAACGGCAGCCATGGTGCCGGATCCGCTGTTATTCAAAACACGTTC |
| AS4C-F2 | GCTTGAGCTTGGGAGGAACCTCAGGCGCTTCAACATTG |
| AS4C-R2 | CAATGTTGAAGCGCCTGAGGTTCCTCCCAAGCTCAAGC |

^a^ Underlined sequences indicate the restriction enzyme sites. Italicized sections indicate the His-tag-encoding sequences. Open boxes indicate the mutated nucleotides.

**Supplementary Table S2. Primer pairs, templates, and restriction enzyme sites used in plasmid construction**

| Plasmid | Primer pair | Template | Method | Restriction enzyme site |
| --- | --- | --- | --- | --- |
| pET26b-*pre-Als* | Als-F + Als-R ^a^ | Genomic DNA |  | *Nde* I-*Eco*R I |
| pET26b-*pre-S225A* | S225A-F + S225A-R | pET26b-*pre-Als* | Quikchange | - |
| pET26b-*pro-Als* | ΔS-F + Als-R ^a^ | pET26b-*pre-Als* |  | *Nde* I-*Eco*R I |
| pET26b-*pro-S225A* | S225A-F + S225A-R | pET26b-*pro-Als* | Quikchange | - |
| pET26b-*mat-Als* | M-F + Als-R ^a^ | pET26b-*pre-Als* |  | *Nde* I-*Eco*R I |
| pET26b-*mat-S225A* | S225A-F + S225A-R | pET26b-*mat-Als* | Quikchange | - |
| pET26b-*pro-AS14C* | ΔS-F + AS4C-R1  AS4C-F1 + AS4C-R2  AS4C-F2 + AS1C-R  AS1C-F + Als-R ^a^  ΔS-F + Als-R ^a^ | pET26b-*pre-Als*  pET26b-*pre-Als*  pET26b-*pre-Als*  pET26b-*pre-Als*  Joined DNA fragments ^b^ | Overlap | *Nde* I-*Eco*R I |

^a^ The primer contained 6 histidines and a stop codon immediately after the gene of interest.

^b^ The joined DNA fragments obtained from the four PCR products above were used as templates in the overlap extension PCR.

**Supplementary Table S3. Charged amino acid content in mature domains of protease Als and other subtilases**

| Subtilase | Organism | pI *^*^* | Number of residues | | | | | | | | Reference |
| --- | --- | --- | --- | --- | --- | --- | --- | --- | --- | --- | --- |
|  |  |  | Total | Negatively charged | | |  | Positively charged | | |  |
|  |  |  |  | D + E | D | E |  | K + R | K | R |  |
| Protease Als  Protease C2  Thermitase  Subtilisin BPN’  Subtilisin Carlsberg  Proteinase K  Halolysin SptA^†^  Halolysin 172P1^†^  Halolysin Nep^†^ | *Thermoactinomyces* *vulgaris* CDF  *Thermoactinomyces* *vulgaris* CDF  *Thermoactinomyces vulgaris*  *Bacillus amyloliquefaciens*  *Bacillus licheniformis*  *Tritirachium album* Limber  *Natrinema* sp. J7  *Haloferax volcanii*  *Natrialba magadii* | 4.26  5.96  7.63  6.80  7.09  8.17  3.56  3.60  3.46 | 279  278  279  275  274  279  287  287  285 | 29  14  15  15  14  18  37  35  48 | 19  13  13  10  9  13  26  23  34 | 10  1  2  5  5  5  11  12  14 |  | 14  10  15  13  13  20  5  3  3 | 10  4  10  11  9  8  1  0  0 | 4  6  5  2  4  12  4  3  3 | This study  (Wang et al., 2015)  (Kleine, 1982)  (Markland and Smith, 1967)  (Jacobs et al., 1985)  (Betzel et al., 1988)  (Shi et al., 2006)  (Kamekura et al., 1992)  (De Castro et al., 2008) |

* The pI values were calculated based on the amino acid sequences of the enzymes.

^†^ Catalytic domain.

**Supplementary Table S4. Ionic pairs of thermitase and protease Als**

| Thermitase ^a^ | Protease Als |
| --- | --- |
| K17-D188  K95-E28  R102-D57  R102-D60  K153-D124  R249-D201  R249-E253  R270-D188  R270-D257  K275-D257 | R102-D57  R102-D60  K153-D124  R249-D201  R249-E253  R270-D188  R270-D257  K275-D257 |

^a^ Ionic pairs are deduced from the crystal structure of thermitase (Teplyakov et al., 1990; Voorhorst et al., 1997)

**REFERENCES**

Betzel, C., Pal, G. P., Saenger, W. (1988). Three-dimensional structure of proteinase K at 0.15-nm resolution. *Eur*. *J*. *Biochem*. 178, 155-171. doi:10.1111/j.1432-1033.1988.tb14440.x

De Castro, R. E., Ruiz, D. M., Giménez, M. I., Silveyra, M. X., Paggi, R. A., Maupin-Furlow, J. A. (2008). Gene cloning and heterologous synthesis of a haloalkaliphilic extracellular protease of *Natrialba magadii* (Nep). *Extremophiles*. 12, 677-687. doi:10.1007/s00792-008-0174-6

Jacobs, M., Eliasson, M., Uhlén, M., Flock, J. I. (1985). Cloning, sequencing and expression of subtilisin Carlsberg from *Bacillus licheniformis*. *Nucleic* *Acids Res.* 13, 8913-8926. doi:10.1093/nar/13.24.8913

Kamekura, M., Seno, Y., Holmes, M. L., Dyall-Smith, M. L. (1992). Molecular cloning and sequencing of the gene for a halophilic alkaline serine protease (halolysin) from an unidentified halophilic archaea strain (172P1) and expression of the gene in *Haloferax volcanii*. *J. Bacteriol.* 174, 736-742. doi:10.1128/jb.174.3.736-742.1992

Kleine, R. (1982). Properties of thermitase, a thermostable serine protease from *Thermoactinomyces vulgaris*. *Acta*. *Biol*. *Med*. *Ger*. 41, 89-102

Markland, F. S., Smith, E. L. (1967). Subtilisin BPN. VII. Isolation of cyanogen bromide peptides and the complete amino acid sequence. *J*. *Biol*. *Chem*. 242, 5198-5211. doi:10.1021/la00041a014

Shi, W., Tang, X. F., Huang, Y., Gan, F., Tang, B., Shen, P. (2006). An extracellular halophilic protease SptA from a halophilic archaeon *Natrinema* sp. J7: gene cloning, expression and characterization. *Extremophiles*. 10, 599-606. doi:10.1007/s00792-006-0003-8

Teplyakov, A. V., Kuranova, I. P., Harutyunyan, E. H., Vainshtein, B. K., Frömmel, C., Höhne, W. E., et al. (1990). Crystal structure of thermitase at 1.4 Å resolution. *J*. *Mol*. *Biol*. 214, 261-279. doi:10.1016/0022-2836(90)90160-n

Voorhorst, W. G., Warner, A., De Vos, W. M., Siezen, R. J. (1997). Homology modelling of two subtilisin-like proteases from the hyperthermophilic archaea *Pyrococcus furiosus* and *Thermococcus stetteri*. *Protein Eng*. 10, 905-914. doi:10.1093/protein/10.8.905

Wang, L., Cheng, G., Ren, Y., Dai, Z., Zhao, Z. S., Liu, F., et al. (2015). Degradation of intact chicken feathers by *Thermoactinomyces* sp. CDF and characterization of its keratinolytic protease. *Appl*. *Microbiol*. *Biot*. 99, 3949-3959. doi:10.1007/s00253-014-6207-4
